# Global Environmental Nontuberculous Mycobacteria and Their Contemporaneous Man-Made and Natural Niches

**DOI:** 10.3389/fmicb.2018.02029

**Published:** 2018-08-30

**Authors:** Jennifer R. Honda, Ravleen Virdi, Edward D. Chan

**Affiliations:** ^1^Department of Biomedical Research and the Center for Genes, Environment, and Health, National Jewish Health, Denver, CO, United States; ^2^Medicine and Academic Affairs, National Jewish Health, Denver, CO, United States; ^3^Division of Pulmonary Sciences and Critical Care Medicine, University of Colorado Denver, Aurora, CO, United States; ^4^Department of Medicine, Denver Veterans Affairs Medical Center, Denver, CO, United States

**Keywords:** nontuberculous mycobacteria, environments, man-made, natural, Hawaii

## Abstract

Seminal microbiological work of environmental nontuberculous mycobacteria (NTM) includes the discovery that NTM inhabit water distribution systems and soil, and that the species of NTM found are geographically diverse. It is likely that patients acquire their infections from repeated exposures to their environments, based on the well-accepted paradigm that water and soil bioaerosols – enriched for NTM – can be inhaled into the lungs. Support comes from reports demonstrating NTM isolated from the lungs of patients are genetically identical to NTM found in their environment. Well documented sources of NTM include peat-rich soils, natural waters, drinking water, hot water heaters, refrigerator taps, catheters, and environmental amoeba. However, NTM have also been recovered in biofilms from ice machines, heated nebulizers, and heater-cooler units, as well as seat dust from theaters, vacuum cleaners, and cobwebs. New studies on the horizon aim to significantly expand the current knowledge of environmental NTM niches in order to improve our current understanding of the specific ecological factors driving the emergence of NTM lung disease. Specifically, the Hawaiian Island environment is currently being studied as a model to identify other point sources of exposure as it is the U.S. state with the highest number of NTM lung disease cases. Because of its geographic isolation and unique ecosystem, the Hawaiian environment is being probed for correlative factors that may promote environmental NTM colonization.

## Background

Nontuberculous mycobacteria (NTM) and their environments are intricately bound. NTM share these environments with humans and domesticated animals and repeated exposure is a well-accepted mode of acquiring these infections. While most casual NTM exposures do not results in disease, those with anatomic lung abnormalities of bronchiectasis and emphysema are particularly predisposed to develop NTM lung disease (NTM-LD). However, in individuals without obvious pre-existing risk factors, it is likely that multiple risk factors – some genetic or other acquired factors – may collude to increase their vulnerability. While there have been case reports and small case series linking genetically identical NTM in patients to their home environment, the specific factors facilitating their acquisition remains poorly characterized. These factors encompass (i) varied sources of infection, (ii) modes of aquisition, and (iii) other physical aspects of the environment such as temperature, humidity, air exchange, surface types, and turbulence created by wind and natural disasters, and (iv) human behaviors, the combination of which are likely to be relatively unique among affected individuals (Honda et al., 2015; Nishiuchi et al., 2017).

Herein, we discuss the traditional environmental niches associated with NTM organisms, but also review the lesser-recognized environmental locales that NTM colonize including non-traditional niches and non-human hosts. We also summarize previous studies that link ecological factors with risk for infection and epidemiological information. Finally, we introduce new, ongoing work to study the particular environmental drivers of NTM emergence in Hawai’i, a geographic location deemed a major hot spot for NTM-LD.

## NTM Origins?

The interaction of humans with their natural and built environments along with changes humans make to their environment (*e.g*., installation of mechanical devices that change environmental temperature and humidity, or others), and differences in the robustness of human health may impact the emergence of infectious diseases. The origins of NTM-LD remain a mystery, but may be intrinsically linked to human interaction with their environments. As soon as early humans learned to prepare small, controlled fires, the light and heat produced gathered people together for social interactions, vastly improved food preparation, and helped communities defend against invasion – activities that significantly lengthened human survival (Chisholm et al., 2016). Because NTM are found in the environment, it is plausible that these bacteria were historically aerosolized from soil with increased fire-making, infecting human lungs already impacted by long-term exposure to campfire smoke. Nonetheless, there is no evidence to show that the number of NTM-LD cases increase after camping or large scale environmental fires. For now, the origins of lung disease caused by NTM organisms remains an area of investigation.

## Global Geography and NTM

The ability of NTM to cause LD and its clinical relevance varies globally. Geographic distribution of NTM species provides information regarding geographic-specific drivers of exposure such as climate, environment, and host factors associated with NTM-LD that would be specific to a global region (Falkinham, 1996; Griffith et al., 2007).

*Mycobacterium avium* complex (MAC) is the most frequently isolated group of NTM species worldwide and the most common organism associated with NTM-LD. MAC consists of various species of slow-growing mycobacteria (SGM) including *M. avium, M. intracellulare*, *M. chimaera*, *M. colombiense*, *M. marseillense*, *M. arosiense*, *M. timonense, M. bouchedurhonense*, and *M. ituriense*. Subspecies of *M. avium* include *avium, silvaticum, hominissuis*, and *paratuberculosis* (Hoefsloot et al., 2013; Johnson and Odell, 2014). Currently, the lowest number of MAC isolates in the world are seen in South America, but this may be because there is little NTM information available from this region (Hoefsloot et al., 2013; Halstrom et al., 2015; Stout et al., 2016). High incidence of NTM-LD due to MAC, *M*. *kansasii*, *M. gordonae*, and *M. malmoense* are observed in Europe, North America and Australia.

Rapid-growing mycobacteria (RGM) including the *Mycobacterium abscessus* and *Mycobacterium fortuitum* groups also contribute to a large proportion of NTM-LD cases globally. The *Mycobacterium abscessus* group is comprised of three subspecies: *M. abscessus* subsp. *abscessus*, *M. abscessus* subsp. *massiliense*, and *M. abscessus* subsp. *bolletii*. In the United States (U.S.), *M. abscessus* complex infections are secondary only to MAC infections, comprising 3-13% of all NTM-LD cases (Lee et al., 2015). The *M. abscessus* group is also commonly observed in patients in East Asia; in Taiwan 17.2% of all clinical NTM isolates belong to the *M. abscessus* group (Lai et al., 2010). The *M. fortuitum* group comprises *M. fortuitum*, *M. peregrinum*, *M. senegalense*, *M. alvei*, *M. houstonense*, *M. neworleansense*, *M. boenickei*, *M. septicum*, and *M. porcinum* (Adekambi and Drancourt, 2004; Schinsky et al., 2004). Besides LD, *M. fortuitum* cause soft tissue, skeletal, catheter-related, and disseminated infections in immunocompromised patients (Brown-Elliott and Wallace, 2002).

### United States

The most commonly occurring NTM species in many parts of U.S. are MAC and *M. kansasii* (Hoefsloot et al., 2013). Nearly 80% of all NTM-LD in the U.S. is due to a species of MAC, followed *by M, kansasii* - the second most common NTM associated with LD (Falkinham, 1996; Griffith et al., 2007). The U.S. states with the highest overall risk for NTM-LD include Hawai’i, California, New York, Louisiana, Pennsylvania, Florida, Oklahoma and Wisconsin (Adjemian et al., 2012a). *M. abscessus* is the most commonly recovered RGM from southeastern parts of U.S. (Halstrom et al., 2015; Stout et al., 2016). For unknown reasons, NTM-LD cases are lowest in North Dakota, South Dakota, Minnesota, Michigan, New Mexico, and West Virgina (Adjemian et al., 2012a).

### Europe and United Kingdom

Higher isolation rates of MAC (44%) are seen in northern Europe as compared to southern Europe (31%) with *M. avium* as the most prevalent species. *M. kansasii* is the predominant NTM species to cause LD in London, United Kingdom. and *M*. *lentiflavum* is most frequently isolated from clinical samples in Crete, Greece (Neonakis et al., 2007; Wassilew et al., 2016). Slovakia, Poland, and the United Kingdom have the highest amount of *M. kansasii* isolates in Europe (Hoefsloot et al., 2013; Wassilew et al., 2016). In contrast, higher isolation rates for *M*. *xenopi* are observed in southern Europe (21%) as compared to northern Europe (6%), but are most commonly isolated in Hungary (46%) (Hoefsloot et al., 2013; Wassilew et al., 2016). RGM are more commonly seen in the United Kingdom and Greece as compared to the rest of Europe (Hoefsloot et al., 2013). While not considered to be a pathogen, *M. gordonae* is most commonly recovered from environmental sources in Canada and Europe (Hoefsloot et al., 2013; Halstrom et al., 2015; Stout et al., 2016).

### Asia

A recent study in China observed that an increase in latitude was associated with higher isolation rates of MAC species (predominantly *M*. *intracellulare*) whereas the number of RGM (most commonly *M. chelonae*) increased with a decrease in latitude (Yu et al., 2016). A similar trend is observed in Taiwan, with higher cases of NTM due to MAC recovered in the north and RGM like *M. abscessus* in the south (Huang et al., 2017). *M. scrofulaceum* and *M. szulgai* are also intermittently found in respiratory specimens from Asia (Simons et al., 2011). Overall, elderly women are disproportionately affected by NTM-LD, but in Saudi Arabia and most of the Persian Gulf countries, elderly men are found to be more affected (perhaps due to a lifetime of extended outdoor exposure) with MAC and *M. abscessus* being the main causative agents (Al-Ghafli and Al-Hajoj, 2017). MAC, *M. simiae*, and *M. marinum* are most commonly observed in NTM-LD individuals in Oman (Al-Ghafli and Al-Hajoj, 2017). In Western Asia, *M. fortuitum* and *M. flavescens* are the most prevalent RGM and SGM organisms, respectively with much higher frequency of NTM in northern Iran (73.2%) (Khaledi et al., 2016). In India, *M. abscessus, M. fortuitum*, and *M. intracellulare* are most commonly isolated from clinical samples (Desikan et al., 2017). *M*. *abscessus* are more widespread in Singapore and Okinawa (Hoefsloot et al., 2013).

### Australia

Similar to the aforementioned geographic areas, LD caused by NTM are also increasingly observed in Australia (Thomson, 2010). MAC is the most commonly isolated NTM in Queensland with *M. intracellulare* comprising nearly 80% of the MAC isolates (Hoefsloot et al., 2013). RGM are the second most common cause of NTM-LD with *M. abscessus* the most commonly recovered from southern Australia (Hoefsloot et al., 2013; Wassilew et al., 2016). Unlike many other regions of the world, NTM are notifiable infections under the Queensland Public Health Act, 2005 which has facilitated the surveillance of potentially highly virulent and transmissible NTM strains (Thomson et al., 2017).

### Africa

Information regarding the causative agents of NTM-LD in Africa are limited and is likely due to the overwhelming burden of tuberculosis in the regions. However, *M. abscessus, M. avium, M. fortuitum*, and *M. nebraskense* are recognized as the most frequently isolated NTM species from clinical samples in Zambia (Monde et al., 2018). *M. gordonae* has been recently found to be highly prevalent in water reservoirs like borehole wells, rivers dams, and tap water (Monde et al., 2018).

## Factors that Help Sustain NTM in the Environment

NTM are slow-growing compared to other types of bacteria, with the ability to form biofilms, resist high temperatures, and grow in marginal environments with low nutrient and oxygen content (Kirschner et al., 1992; Falkinham, 2009). However, cell surface hydrophobicity is the major driver sustaining NTM in the biofilms of both natural waters and man-made drinking water distribution systems, hospitals, and household plumbing. Due to their repulsion to water, NTM are found in aerosolized particles present above natural water bodies, showerheads, humidifiers, hot tubs and spas as well as in biofilms that form in these places (Falkinham, 2013). Environmental factors such as high humidity levels and high evapotranspiration (movement of water from land to the atmosphere) rates are known to be associated with an increased risk of NTM infection in susceptible individuals (Adjemian et al., 2012a). This is particularly translatable to the recovery of NTM during different seasons. For example, the species of NTM isolated from municipal water distribution systems in Brisbane, Australia differed in the samples collected in summer as compared to those collected in the winter months with higher numbers of *M. gordonae, M. kansasii, M. abscessus, M. mucogenicum*, and MAC isolated in the winter (Thomson R. M. et al., 2013).

Other environmental factors have a profound impact of NTM viability. For example, NTM have been isolated from water bodies with moderate salinity (1–2% NaCl) like estuaries (Chesapeake Bay) (Kirschner et al., 1992; Falkinham, 2009). But in a separate study, reduced numbers of NTM isolates were observed when water salinity exceeded 2% (Gruft et al., 1981). NTM also favor environments with acidic pH. Humic and fulvic acids and acidic brown water swamps along the southeastern coast of the U.S. support high numbers of MAC (Falkinham, 2009, 2013). Pine forest (boreal rich) and peat rich soils, brackish marshes, and drainage water are also rich in NTM (Kirschner et al., 1992; Falkinham, 2009). Minerals widely found in clay soils such as kaolin and dust have also been demonstrated to facilitate the growth of *M. abscessus* (Malcolm et al., 2017).

## Typical Environmental Habitats of NTM

*M. avium*, *M. fortuitum*, *M. chelonae*, *M. kansasii*, *M. gordonae*, and *M. xenopi* are the NTM species most commonly found in water distribution systems, water bodies including lakes, rivers and streams as well as soil and dust (Falkinham, 2009; Ulmann et al., 2015). Falkinham et al. (2008) was the first to demonstrate that the *M. avium* isolated from a patient with NTM-LD had a clonal relationship with the *M. avium* isolated from her home showerhead biofilm. Falkinham also first reported identical NTM DNA fingerprints from patients’ sputa and matched shower water isolates, shedding light on the paradigm that inhalation of aerosols while showering is a likely mode of NTM acquisition (Falkinham, 2011). Similar reports are observed from Japan where MAC isolates recovered from bathtub inlets and showerheads showed identical pulse-field gel electrophoresis profiles when compared to their respective clinical isolates (Nishiuchi et al., 2009; Ichijo et al., 2014). Thus, showerhead biofilms remain one of the most frequently sampled environmental sources used to describe the presence of NTM globally. In an interesting turn of events, early methods such as hybridization probes and multiplex 16S rRNA gene PCR methodologies identified *M. intracellulare* in households and potable water (Tichenor et al., 2012; Whiley et al., 2012). However, Falkinham et al. (2008) and Wallace et al. (2013) reanalyzed environmental household water and biofilm isolates originally identified as *M. intracellulare* by sequencing the 280 bp 16S to 23S internal transcribed spacer region and discovered that these isolates were instead *M. chimaera* (Thomson, 2010; Falkinham, 2011; Koh W. J. et al., 2012; Tichenor et al., 2012). Using the same sequencing method, isolates originally called *M. avium* were confirmed as *M. avium*. Thus*, M. chimaera* is now widely recognized in water biofilms, while *M. intracellulare* is found to be absent from them. A clue to their sources may come from a study conducted in American Samoa where *M. intracellulare* was identified in roof-harvested rainwater (Kirs et al., 2017) and soil (Honda et al., 2016).

NTM found in households are likely piped into home plumbing systems from public utility sources where biofilms are commonly formed. However, NTM are also known inhabitants of natural freshwater ecosystems. Of two recreational lakes, RGM were the dominant NTM, but a diversity of other mycobacteria were found in high density in the water column, air-water interface, sediment, and in association with benthic algae growing on plants and fine sediment using quantitative real-time PCR and the MiSeq Illumina platform (Roguet et al., 2016). Yet, NTM remain seldomly recovered from well and groundwaters (Martin et al., 1987).

Nosocomial NTM lung infections have been reported in the literature. MAC species have been detected in hospital potable hot water distribution systems, hospital tap water used for dialysis, and in water used to prepare medical solutions, highlighting their propensity to stick to piped surfaces (Bolan et al., 1985; Safranek et al., 1987; Hector et al., 1992). After a significant increase in NTM-positive sputa was observed from patients referred to respiratory wards in Rome, an infection control investigation revealed a massive presence of NTM in the hospital water network (D’Antonio et al., 2016). In another study, 83% of U.S. dialyses centers examined showed NTM in municipal water supplies (Carson et al., 1988). More recently, global outbreaks of *M. chimaera* associated with heater-cooler units used during open-heart surgery have provided unique challenges for the medical community (Sax et al., 2015; Schreiber et al., 2016; Marra et al., 2017). Investigations point to model-specific designs in air flow direction, location of cooling ventilators, and the continuous cooling of unit water tanks significantly increased the risk of disseminating colonized *M. chimaera* into the air of operating rooms (Kuehl et al., 2018). Of the first thirty cases affected by this outbreak in the United Kingdom, 60% (18/30) died at a median of 30 months after initial surgery (Scriven et al., 2018). Poor disinfection and resistance to glutaraldehydes have been highlighted in pseudo-outbreaks of *M. abscessus* subsp. *bolletii* in bronchoscopes, endoscopes, and disinfection units (Guimaraes et al., 2016). In other areas of the hospital, patient accessible ice machines have been shown to be laden with NTM, particularly *M. paraffinicum* and *M. fortuitum* (Gebo et al., 2002; Wang et al., 2009).

*M. fortuitum* skin infections have been associated with pedicure-associated whirlpool footbaths in California and Georgia nail salons (Winthrop et al., 2004). Skin infections due to other NTM such as *M. chelonae* have been linked to contaminated water used for diluting tattoo ink and to unsterilized instrumentation (Mudedla et al., 2015). Dental unit water lines have also been shown to harbor a variety of environmental NTM species (Schulze-Robbecke et al., 1995), but remain as unproven sources of NTM lung infection. MAC-associated hypersensitivity pneumonitis (HP) has been linked to exposure to warm, bubbly water found in rarely cleaned hot tubs and spa baths (Embil et al., 1997; Sugita et al., 2000; Rickman et al., 2002). However, MAC has also been isolated from cold water sources including swimming pool water. In fact, lifeguards with long-term exposure to indoor swimming pool aerosols are susceptible to work-associated exposures and are at increased risk for MAC-associated HP (Rose et al., 1998; Koschel et al., 2006). Besides tap water, a Dutch group found NTM in swimming pools and whirlpool water (Havelaar et al., 1985). NTM-associated HP has also been linked to occupational exposures to aerosols produced through manipulation of metalworking fluids (Bernstein et al., 1995; Wilson et al., 2001).

Soil is also a widely recognized environmental niche for NTM. NTM patients’ potting soils yielded NTM that were identical by DNA fingerprinting to the NTM isolates from the same patients’ lungs (Ichiyama et al., 1988; De Groote et al., 2006). Moreover, *M. avium* subsp. *hominissuis* (MAH) was predominant in soil and dust, but not identified in German water and biofilm samples by culture (Lahiri et al., 2014). A study in Iran found that 6-15% of soil samples compared to 10–27% of water samples collected from the suburbs of Tehran had NTM isolated by culture with *M. farcinogens* and *M. fortuitum* being the most common species (Velayati et al., 2014). Furthermore, the risk for acquiring NTM is significantly higher in communities engaged in occupations that generate aerosols and are exposed to soil for a longer time (*e.g.*, agriculture, mining, landscaping, and tunnel work) as compared to communities that have a limited exposure to soil (Hamada et al., 2016). In West Harima, Japan, NTM-LD was associated with natural resource activities, construction, mining, and soil exposure (Hamada et al., 2016). In a separate study, *M. chelonae, M. fortuitum*, and *M. kansasii* were identified in 85% of the alpine and subalpine soil, peat, humus, porous ricks, mosses, and wood examined suggesting NTM thrive in mountain ranges and elevations (Thorel et al., 2004). Rare and unique NTM species have also been described in polluted soils of Hawai’i where they functioned as polycyclic aromatic hydrocarbon pollutant-degrading organisms (Hennessee et al., 2009).

## NTM in the Kitchen

NTM have been reported in kitchen sink biofilms as well as household refrigerator taps and home ice machines (Ichijo et al., 2014). MAC organisms have been found to colonize point-of-use filters used to filter tap water including carbon filers impregnated with silver (Rodgers et al., 1999; Hamilton et al., 2017). Due to the appearance of disseminated *M. avium* infections during the height of the HIV-AIDS epidemic, two studies tested for the presence of NTM in foods consumed by HIV-infected patients. PCR typing revealed 29 different mycobacterial isolates in 21% (25/121) of food samples tested; 41% of the 29 samples (*n* = 12) were *M. avium* (Yoder et al., 1999). One of the clinical *M. avium* isolates was identical to a food isolate, suggesting food as a potential source of *M. avium* infections. In the second study, water, food and soil samples from 290 homes of HIV-infected patients were tested for mycobacteria using DNA probes, serotyping, and multi-locus enzyme electrophoresis and compared to clinical isolates (Yajko et al., 1995). Soil, rather than water sources, were found to harbor more *M. avium*. While not considered a food-borne illness, *M. avium* subspecies DNA was also identified in raw meats (Lorencova et al., 2014). In contrast, smoked fish products did not show NTM, but 12% of samples collected from pond fish (4%), retail sold fish (61%), and frozen fish (91%) contained NTM DNA (Lorencova et al., 2013). Although unproven, the acid-resistant NTM organisms may remain viable in the stomach where food is consumed and digested. Evidence suggests patients with the nodular bronchiectatic form of NTM-LD have a high prevalence of increased esophageal acid exposure and gastroesophageal reflux disease was found to be significantly associated with RGM organisms (Winthrop et al., 2010; Koh E. et al., 2012).

## NTM in Non-Canonical Environments

Besides their well-known habitation in the numerous sources detailed above, rare NTM species (*e.g.*, *M. algericum, M. arabiense, M. heraklionense*) have been cultured and identified from primary sludge samples of water treatment plants even after decontamination (Makovcova et al., 2015). *M. avium, M. gordonae*, and *M. flavescens* have been also been identified in non-traditional water sources including untreated, drinking well water in rural areas of Montana as well as in treated municipal wastewater from arid regions (Richards et al., 2015; Amha et al., 2017). Qualitative assessments were used to determine the risk of MAC exposure in Queensland, Australia, an area that utilizes rainwater catchment systems. Untreated rainwater is commonly used for showering, car washing, toilet flushing, and food preparation (Hamilton et al., 2017). But in this study, rainwater used for drinking presented the greatest risk for MAC infection; yet the species of MAC responsible was not reported. In most cases, disinfection methods to remove potential pathogens from these water sources is a decision left up to the household and can range from no disinfection methods to point-of-use filters, UV irradiation, solar disinfection, chlorine or a combination of them. More work is needed in these areas to determine the impact of using different disinfection methods to reduce exposures in NTM patients who use these water source types.

NTM have also been detected, albeit, rarely in cobwebs above hen nests, soil fertilized with chicken droppings, and moss but more commonly in dust from vacuum cleaners, and air conditioners (Nishiuchi et al., 2009; Kaevska et al., 2011). Of particular significance to smokers is the recovery and identification of *M. avium* from cigarettes (Eaton et al., 1995). While not directly shown to cause LD, MAC has been identified from clothing washed during laundry cycles suggesting laundry water maybe an unintentional source of household NTM (Yajko et al., 1993). Finally, *M. avium* was detected in samples of condensation water formed from the coagualation of steam in three different rooms inside the Russian space station, Mir (Kawamura et al., 2001).

## The “Environmental Macrophage” and NTM

Amoeba are free-living, freshwater associated protozoans that are ubiquitously found in water systems often cohabited by NTM. Many species of amoeba phagocytose free-living bacteria and feed on them; however, some NTM are able to resist and evade degradation. In particular, most species of MAC, including *M. avium*, *M. intracellulare*, *M. chimaera*, *M. colombiense*, *M. arosiense*, *M. marseillense*, *M. timonense*, and *M. bouchedurhonense* reside within free-living *Acanthamoeba polyphaga* and their exocysts as well as *A. castellanii* found in potable water (Cirillo et al., 1997; Taylor et al., 2009; Ben Salah and Drancourt, 2010). Delafont et al. (2014) demonstrated amoeba-mycobacteria associations in drinking water networks in a year-long sampling study. Nearly 88% of amoeba including the genera *Acanthamoeba, Vermamoeba, Echinamoeba*, and *Protacanthamoeba* recovered from drinking water were found to contain *M. llatzerense* and *M. chelonae* (Delafont et al., 2014). NTM cultured in amoeba also show increased resistance to antibiotics and enhanced virulence compared to NTM grown in chickens and mice (Cirillo et al., 1997; Falkinham et al., 2001).

## Evidence for Animal and Insect Reservoirs for NTM Associated with Extrapulmonary Infections

NTM infections in mammals occur sporadically and are rarely transmissible between animals and seldomly considered *bone fide* zoonotic diseases. Except under particular scenarios, NTM infections are also generally not transmissible from human to human (Bryant et al., 2016). Nonetheless, NTM have been known to infect animals such as chickens and quails (Meissner and Anz, 1977; Morita et al., 1999). Drug-susceptible *M. fortuitum* and *M. abscessus* were identified in a cutaneous lesion on the snout and nostrils of a captive *Trichechus inunguis* (manatee) in the Amazon (Reisfeld et al., 2018). In captive non-domesticated hoofed animals and in immunosuppressed dogs and cats, *M. avium* has been reported as disseminated disease (Thorel et al., 2004). With the potential for cross species infection or transmission in the Serengeti, tissues from wildlife species and indigenous cattle were probed for mycobacteria, revealing *M. intracellulare* as the most frequently isolated species, followed by *M. lentiflavum, M. fortuitum*, and *M. chelonae/abscessus*. MAC organisms were also detected in animal feces and huts from pastoral Uganda (Kankya et al., 2011). Because *M. avium* isolates from pigs showed shared genetic characteristics to *M. avium* isolated from humans, pigs have been theorized as potential sources of infection (Bono et al., 1995). Among 1,249 mandibular lymph node samples collected from the wild boar, *Sus scrofa*, between 2007 and 2011 in Spain, *M. chelonae* and *M. avium* represented 61 and 11% of the NTM isolates (Garcia-Jimenez et al., 2015). In a separate study, a low degree of similarity between MAH isolates from Japanese patients and local pigs was found, while there was a high degree of similarity between European patient and pig isolates, suggesting geographic distinctions (Iwamoto et al., 2012).

NTM infections are among the most common chronic disease of aquatic animals (Gcebe et al., 2018). After their original discovery in 1897, NTM organisms continue to cause disease in sea bass, mullets, and amberjacks that live in temperate zones (Lescenko et al., 2003). The most common NTM pathogens of fish include *M. chelonae* (sea horses), *M. avium*, and *M. fortuitum*. *M. marinum* remains the most typical NTM found in aquatic environments and often coinhabit on shrimp, frogs, eels, oysters, and shellfish (Piersimoni and Scarparo, 2009). *M. marinum* is one of the only known species of NTM that grow in waters with high salt concentrations (>3% NaCl) (Kirschner et al., 1992; Falkinham, 2009). In some cases, *M. marinum* cause extrapulmonary infections and cutaneous “fish tank granulomas” in humans. Controlling NTM infections in the aquaculture setting is difficult, relying only on destruction of the infected stocks in the absence of effective treatments (Gcebe et al., 2018). *Carassius auratus* (goldfish) have been evaluated as a novel *in vivo* model to study the pathogenesis of *M. marinum*. After intraperitoneal administration of 10^2^ and 10^9^ CFU of *M. marinum* organisms, an acute and chronic infection was respectively observed with high recovery of NTM from inoculated animals (Talaat et al., 1999).

Although seldom reported, reptiles and insects can potentially carry pathogenic NTM. *M. chelonae* was originally isolated from the lungs of sea turtles in 1903 (Männikkö, 2011), *M. intracellulare* has been identified in a rusty monitor reptile with lung nodules (Friend and Russell, 1979), and *M. szulgai* has been isolated from a crocodile showing tuberculosis-like lung lesions (Gcebe et al., 2018). In a separate case, *M. gordonae, M. avium*, and *M. kansasii* were isolated off cockroaches from a South Taiwan hospital (Pai et al., 2003).

*Mycobacterium ulcerans* can infect skin and subcutaneous tissues developing into non-ulcerated nodules or lesions. Although *M. ulcerans* has not yet been cultured from the environment, its DNA has been detected in low levels in suspended solids/water residues and soil. High concentrations of *M. ulcerans* DNA are observed in the feces of Australian ringtails and brushtails possums residing in locations where human cases were reported. These findings suggest possums are naturally infected and are potential environmental reservoirs (Fyfe et al., 2010).

## Studies Linking NTM Ecology with Available Epidemiological Data

To understand the environmental risk factors for infection, most studies described above have either probed for the occurrence of NTM in various environmental sources or in pulmonary samples from various geographic areas. Yet, the number of studies that have overlaid and integrated ecological and geographic information with epidemiological information of NTM-LD risk are scant. However, an exciting recent study has enhanced our understanding of how ecology relates to risk for NTM infection. In Colorado, soil acidity, low manganese concentrations, and silt were significantly associated with increased disease risk for NTM (Lipner et al., 2017). This same study also identified high-risk clusters of NTM-LD and high-risk watershed locations in the same geographic region using spatial scanning methods. Another study from Queensland, Australia (2001–2010) investigated the associations between climate, soil characteristics, land use, demographic, and socio-economic variables with spatial patterns of NTM infection (Chou et al., 2014). High risk clusters of *M. kansasii, M. intracellulare*, and *M. abscesses* infections were found to be associated with areas of high agricultural, mining, and tourism activity.

Higher rates of NTM lung infections have been associated with oceanic coastlines, accounting for 70% of annual NTM cases in the U.S. (Strollo et al., 2015). Statistically significant increases in NTM identification have been reported in the U.S. Affiliated Pacific Islands (USAPI) including American Samoa, Guam, Northern Marina Islands, Palau, Marshall Islands, and the Federated States of Micronesia (Lin et al., 2018). After analyzing respiratory cultures submitted for species identification between 2007 and 2011 at a clinical reference laboratory, the overall period prevalence of NTM isolation in this study was 106 cases/100,000 persons. The authors acknowledge that the prevalence of NTM isolation varied by island nation and may be related to urbanization. The lowest period prevalence of NTM isolation (22 cases/100,000 persons) was reported in American Samoa with 87.2% of patients living in high urban areas. The highest period prevalence (164/100,000 cases) was observed in respiratory samples from the Federated States of Micronesia which also showed the lowest percentage of urban dwellers (22.4%).

Of interest, the first nationwide population-based analysis on the prevalence of NTM-LD found that Hawai’i had the highest period prevalence (1997–2007) of any U.S. state with 396 cases/100,000 persons among persons > 65 years-old (Adjemian et al., 2012b), a rate that is four times greater than the national average. Using national Medicare claims, U.S. Census data, as well as U.S. Geological and Forest Services environmental and climatic data, high and low risk U.S. counties were identified (Adjemian et al., 2012a). High-risk regions for NTM were noted in particular geographic areas of Hawai’i. Higher daily evapotranspiration levels, higher number of surfaces covered by water, higher soil copper and sodium, and lower manganese levels were characteristics of high risk areas. More ominous, Hawai’i also shows the highest, national age-adjusted mortality rates from NTM-LD (Mirsaeidi et al., 2014).

**FIGURE 1:**
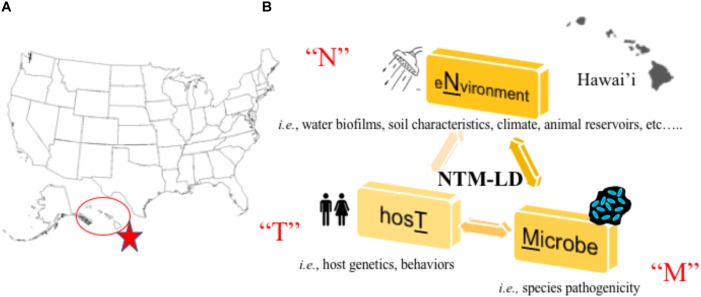
**(A)** Hawai’i is a hot spot for NTM lung disease in the United States (Rudis, 2018). **(B)** Our research program aims to study the “N-T-M” specific factors that contribute to lung disease by studying the environmental, host, and microbial factors that facilitate NTM lung disease emergence in Hawai’i.

In a recent large-scale study, researchers partnered with a network of citizen scientists to collect showerhead biofilm samples from 606 households across 49 of 50 U.S. states to simultaneously superimpose the identities of showerhead microbes over geographic areas where NTM-LD is most prevalent (Gebert et al., 2018). Multiple concluding findings were reported that reinforce known paradigms. First*, Mycobacteria* was the most abundant group of bacteria identified in showerhead biofilms by *16S* rRNA gene sequencing, corroborating prior studies (Feazel et al., 2009; Thomson R. et al., 2013). Next, the most frequently identified NTM species by *hsp65* gene sequencing were MAC, *M. abscessus*, and the *M. fortuitum* complex which are also the most problematic NTM for patients. As already observed (Garcia et al., 2013; Ovrutsky et al., 2013), mycobacteria often co-occurred with free-living amoeba. Second, similar to other studies (Feazel et al., 2009), homes using municipal treated water showed twice more mycobacteria than homes using water from wells and the former contained higher chlorine and iron concentrations. Third, by overlapping these data with previously reported epidemiological information, this study is the first to show showerhead-associated mycobacteria with the potential to cause pathogenic LD are indeed found in U.S. households in regions with the highest number of NTM-LD cases including Florida, southern California, and northeast states. It is clear mycobacterial lineages showed distinct geographic distributions. Finally, supporting prior work, this study confirms Hawai’i as the state with the most abundant mycobacterial showerhead biofilms and show these are predominated by MAC and *M. abscessus* organisms. Undoubtedly, Hawai’i is a U.S. hot spot for NTM-LD (**Figure 1A**) and the islands’ unique characteristics may contribute to greater exposures.

## Movement to Collectively Understand NTM Ecology, Epidemiology, and Organism Virulence in the Hawaiian Island Environment

We have previously shown increased prevalence of NTM infection in Hawai’i using patient laboratory data from a representative population of in-state residents enrolled in a closed healthcare system (Adjemian et al., 2017). Indeed, the most frequently isolated species was MAC, followed by *M. fortuitum* that was more common among Vietnamese and Korean patients, while *M. abscessus* was more associated with Japanese and Filipino patients. In this study, Native Hawaiian/Other Pacific Islander populations were less likely to have NTM infection than other racial/ethnic groups examined. A logical hypothesis is that native populations inhabited the islands for a longer period of time, evolving and developing resistance to infection.

We have also previously demonstrated not only the overwhelming presence of MAC in both clinical samples in respiratory specimens from Hawai’i, but also in water biofilm samples collected from households in Hawai’i (Honda et al., 2016). Using partial *rpoB* gene sequencing, *M. avium* was not identified in any of the samples examined, providing a contrast to previous studies from the continental U.S. *M. intracellulare* was found in 27% (4/15) SGM respiratory specimens examined and a soil sample. While absent from soil, *M. chelonae* was significantly more common in kitchen sink biofilms (9/35, 35%) compared to bathroom sink biofilms (3/34, 9%) and *M. abscessus* was found equally in kitchen (5/34, 15%) and bathroom sink (4/30, 13%) biofilms. *M. porcinum* was also frequently recovered from showerhead biofilm samples, a species normally associated with swine, but also a cause of human infections (Wallace et al., 2004; Brown-Elliott et al., 2011; Perez-Martinez et al., 2013). This observation may be particularly important in Hawai’i, which has a sizeable feral pig population (Hess et al., 2006). Importantly, we identified *M. chimaera* as the predominant species of MAC in these islands; in fact, *M. chimaera* was recovered from 56% of the 75 environmental samples tested and 67% of ten clinical isolates tested (Honda et al., 2016). By comparison, of 8,800 isolates analyzed using *rpoB* gene sequencing in 26 months at National Jewish Health, only 6% were *M. chimaera* (Dr. Max Salfinger, National Jewish Health, personal communication). Taken together, *M. chimaera* is emerging as a major NTM species of interest in Hawai’i, underscoring the need for further studies to define the drivers for NTM emergence there and in other Pacific Islands.

In new and on-going work to understand the Hawai’i specific environmental, host, and NTM factors that contribute to NTM-LD emergence (**Figure 1B**), we are synergizing environmental and human behavioral/genetic findings with microbiological and NTM genomic data in dynamic statistical, spatial/temporal models to uncover not only disease drivers, but also potential points of intervention to prevent future infections. Isolates collected through this work will also be applied in future studies of NTM virulence. To accomplish this, a complementary team has been formed including a NTM microbiologist born and raised in Hawai’i with significant ties to the local community, a mycobacterial pulmonologist, epidemiologist, earth geochemist, climatologist, microbial ecologist, volcanic scientists, ecological modelers, local clinicians, and a team of microbial genomic and computational scientists as well as Hawai’i residents, high school/college students, their mentors, and local pig hunters.

## Future Directions

Future studies should investigate the: (i) factors that drive the relative absence of NTM from seawater, but association with higher humidity and associated biofilms collected from different areas of the world, (ii) NTM prevalence and diversity in areas of the world with endemic tuberculosis, (iii) role of animal and protozoal reservoirs in the maintenance and spread of environmental NTM, and (iv) contributions of temperature, evapotranspiration, and air pollution including climatic determinants of disease. In the meantime, we spotlight the NTM crisis in Hawai’i as a useful model system to understand NTM transmission and disease dynamics. New information gathered from this work will then be used and applied to study NTM-LD in other areas of the world where NTM-LD is prevalent and emergent.

## Author Contributions

JH conceived the project. JH, RV, and EC wrote and edited the manuscript.

## Conflict of Interest Statement

The authors declare that the research was conducted in the absence of any commercial or financial relationships that could be construed as a potential conflict of interest.
